# Dietary overlap and selectivity among mountain steppe river fish in the United States and Mongolia

**DOI:** 10.1002/ece3.10132

**Published:** 2023-05-21

**Authors:** Mario Minder, Emily R. Arsenault, Mark Pyron, Amarbat Otgonganbat, Bud Mendsaikhan

**Affiliations:** ^1^ Department of Biology Ball State University Muncie Indiana USA; ^2^ Kansas Biological Survey and Department of Ecology and Evolutionary Biology University of Kansas Lawrence Kansas USA; ^3^ Professional Biological Society of Mongolia Ulaanbaatar Mongolia; ^4^ Institute of Geography and Geoecology, Mongolian Academy of Sciences Ulaanbaatar Mongolia

**Keywords:** diet, invasive, mountain steppe, overlap, selectivity

## Abstract

Lotic systems in mountain regions have historically provided secure habitat for native fish populations because of their relative isolation from human settlement and lack of upstream disturbances. However, rivers of mountain ecoregions are currently experiencing heightened levels of disturbance due to the introduction of nonnative species impacting endemic fishes in these areas. We compared the fish assemblages and diets of mountain steppe fishes of the stocked rivers in Wyoming with rivers in northern Mongolia where stocking is absent. Using gut content analysis, we quantified the selectivity and diets of fishes collected in these systems. Nonnative species had more generalist diets with lower levels of selectivity than most native species and native species had high levels of dietary specificity and selectivity. High abundances of nonnative species and high levels of dietary overlaps in our Wyoming sites is a cause of concern for native Cutthroat Trout and overall system stability. In contrast, fish assemblages characterizing Mongolia mountain steppe rivers were composed of only native species with diverse diets and higher selectivity values, suggesting low probability for interspecific competition.

## Graphical abstract

The introduction of nonnative species can influence the dietary characteristics of native species. We compared fishes in rivers with and without the presence of nonnative species to compare the diets of native species. We found nonnative species had more generalist diets and greater dietary overlaps compared to native species.

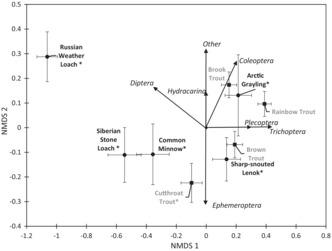

## 1 INTRODUCTION

Lotic systems in mountain regions have historically provided secure habitat for native fish populations because of their relative isolation from human settlement and lack of upstream disturbances (Adams et al., 2001; Isaak et al., 2016). However, rivers of mountain ecoregions are currently experiencing heightened levels of anthropogenic disturbance (Hofmann et al., 2015; Leu et al., 2008; Wohl, 2006). In the expansive mountain steppe ecoregions characterizing the mountain regions of western United States (US) and northern Mongolia, multiple anthropogenic pressures have the potential to impact endemic fish species (Kaus et al., 2019). In the mountain steppe rivers of Mongolia, fishes started to be exposed to nonnative species (Mendsaikhan et al., 2017) and impacts caused by mining and free‐range livestock grazing (Chalov et al., 2012). In western US mountain steppe rivers, fishes are impacted by beaver removal, habitat alterations from mining activities, and the introduction of nonnative fishes (McKelvey et al., 2016; Wohl, 2006). Stocking hatchery‐raised native and nonnative salmonid species can be detrimental to wild fishes and associated assemblages due to increased competition for food, potential for hybridization and predation of native fishes (Seiler & Keeley, 2009).

Gut content analysis is a low cost and informative method of dietary analysis which provides details on a range of dietary information (Baker et al., 2014; Declerck et al., 2002; Pilger et al., 2010). By determining the degree of dietary overlap among species, we can predict potential impacts of introduced species on native species (Declerck et al., 2002; Pilger et al., 2010; Sampson et al., 2009). Identification of specific dietary items, though the use of gut content analysis, also allows for the calculation of selectivity indices identifying diet item preferences, key dietary components and how these preferences may change based on biotic (seasonal fluctuations in diet populations, interactions with other fish species, etc.) and abiotic (elevation, temperature, flow, etc.) factors (Hilderbrand & Kershner, 2004; Lowe et al., 2000; Mischke et al., 2003; Nakano et al., 1999).

The research presented here is a small part of a larger macrosystem ecology project comparing rivers in the Western United States and Mongolia which contrasted in their level of human impact. The aim of this project was to compare the fish assemblages and diets of mountain steppe fishes of the stocked, heavily managed rivers of the western United States and rivers in northern Mongolia where stocking is absent. Understanding how diets differ in different fish assemblages based on the presence of stocked nonnative fishes allows us to predict potential impacts on recently altered systems. We hypothesized that (1) in Wyoming, where native and nonnative species overlap, nonnative species will have generalist diets without high levels of selectivity and native species will have specialized diets with higher levels of selectivity; and (2) in Mongolia where nonnative fishes are not well established, native fishes will have specialized diets and high dietary selectivity, and (3) Wyoming fish assemblages will have higher dietary overlaps compared to those of Mongolia because of the co‐occurring of native and nonnative fish in Wyoming.

## 2 METHODS

### 2.1 Study area

**FIGURE 1:**
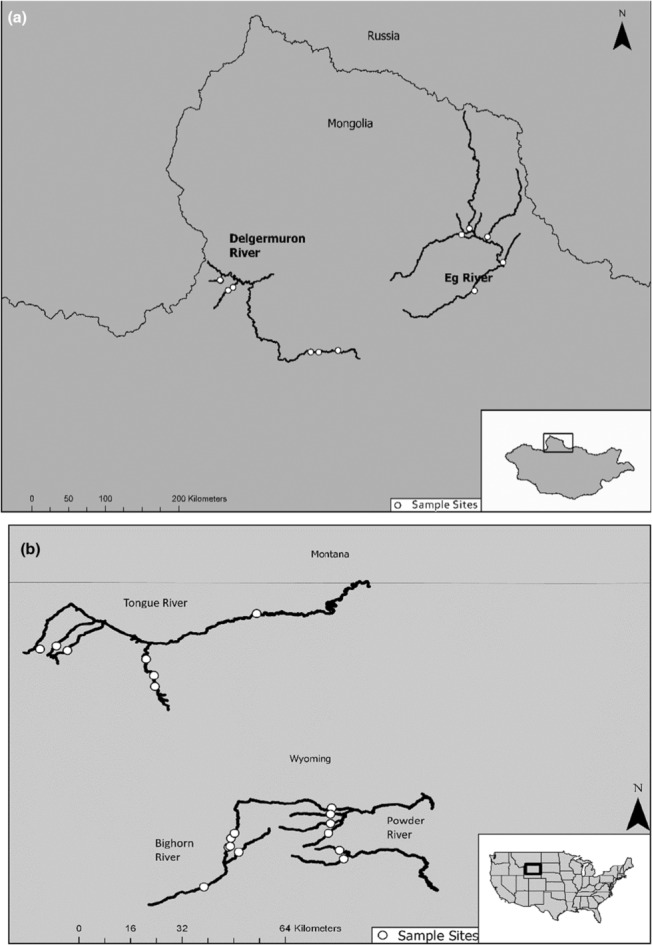
Two maps displaying our study sites in Wyoming (Powder, Bighorn, and Tongue rivers) and Mongolia (Delgermuron and Eg rivers). The rivers are highlighted with white points representing the sample sites used for the project.

We sampled 20 sites in summer 2017 in three Yellowstone River watersheds (Bighorn, Powder, and Tongue Rivers) in the Wyoming Mountain steppe, and 12 sites in two Selenge River watersheds (Delgermörön and Eg rivers) (Figure 1). Sites were chosen to maximize variability in hydrogeomorphology and ensure that sites accurately represented the broad geographic ecoregions that we sampled. Sites were selected as part of a larger macrosystem ecology project using the GIS‐based tool RESonate to characterize river segments using valley‐scale hydrogeomorphic variables (Williams et al., 2013). Specific stream metrics for all of our sample sites can be found in Appendix A, and geomorphologic variables for these areas can be found in more detail in Shields et al. (2021), a related project that was conducted under the same broader macrosystems project.

### 2.2 Fish and benthic invertebrate collections

At each site, fishes were collected from reaches measuring 20 times the average wetted width of the stream (Patton et al., 2000). Fishes were first collected using one‐pass backpack electrofishing (ETS model: ABP‐2) supplemented with hook and line and seining following electrofishing in areas where water depth or conductivity may have impacted electrofishing, following American Fisheries Society standard collection protocols (Bonar et al., 2009). To assist in areas where electrofishing success is non‐optimal due to low conductivity, supplemental hook and line and seines were used. All collected fishes were identified to species, weighed (g), and measured for standard length (mm). When available, up to 10 fish for each species at each site were randomly selected and sacrificed for gut analysis. Stomachs were removed and preserved in 10% formalin for later analysis. For fishes lacking a true stomach, the anterior portion of the gut to the first bend was used as a proxy (Rybczynski et al., 2008). For all fishes, only the stomach or anterior portions of the gut was examined in an effort to minimize bias caused by digestibility of diet items (Sutela & Huusko, 2000).

A quantitative survey (abundance per m^2^) of benthic invertebrates based on methods from Minder et al. (2020) was conducted at all study sites prior to fish collection. We collected benthic invertebrates to determine the proportional environmental abundance of each diet item for diet selectivity analyses. Benthic invertebrates were collected from three microhabitats (riffles, runs, and pools) using a Surber net, Hess sampler and a modified corer sampler (0.09, 0.03, and 0.06 m^2^, respectively, mesh size 350 μm) and five samples were collected by each microhabitat resulting in a total of 15 samplers per site (Minder et al., 2021). All samples were preserved in ethanol (70%) in the field and sorted and identified in the laboratory using a number of keys for aquatic macroinvertebrates (Merritt et al., 2008; Thorp & Covich, 2009). After processing, samples were grouped by sites to calculate mean invertebrate abundance per site.

### 2.3 Diet analyses

Gut analysis followed procedures based on previously published works (Minder et al., 2020, 2021). Guts (esophagus to pyloric valve) were evacuated of all contents in the laboratory, and contents were examined under a dissecting scope. All items were identified to family using keys and grouped by order (Merritt et al., 2008; Thorp & Covich, 2009). Orders that represented <1% of the total number of diet items among all species were grouped into a single category, referred to as “Other.” Numerical abundances of each diet item were recorded, and average proportional abundances were calculated for each order.

Calculations of frequency of occurrence (FO), and mean prey abundance (Ni) were used to quantify diets of individual fishes. FO was calculated as:

$$\mathrm{FO}=\left[\frac{F_{i}}{P}\right]\times 100$$

where FO is the occurrence of a prey item $F_{i}$ divided by the number of non‐empty guts (*P*). The metric FO describes the percentage of individuals that have consumed a specific food item. While this metric does not provide details on amounts of items consumed, it is robust to limitations of other diet analysis challenges such as differences in prey condition and presence of unidentifiable tissues (Baker et al., 2014; Buckland et al., 2017).

Mean prey abundance (*N*_*i*_) was used to compare feeding behavior and diet composition among fishes (Macdonald & Green, 1983). *N*_*i*_ was calculated as:

$$N_{i}=\frac{1}{P}\times \left(\sum \left[\frac{N_{\mathrm{ij}}}{\sum N_{\mathrm{ij}}}\right]\right)$$

where *N*_*i*_ is the mean number of prey *i* consumed, *N*_*ij*_ is the number of prey *i* in a single predator *j*, and *∑N*_*ij*_ is the sum of all the prey in a single predator gut *j*.

Dietary behavior was quantified with Chesson's α selectivity index (Chesson, 1978):

$$\alpha =\frac{\left(r_{i}/p_{i}\right)}{\sum \left(r_{i}/p_{i}\right)}$$

where *r*_*i*_ is the proportion of the diet item consumed by an individual fish, *p*_*i*_ is the proportional environmental abundance of the diet item at the capture site, and *n* is the number of prey item categories present. If α = 1/*n*, the item in the diet is equal to its proportion in the environment, and we can assume that the item has been randomly selected. If α > 1/*n*, then the diet item has been positively selected for, and if α < 1/*n*, then that diet item has been avoided. Environmental abundances for diet items were calculated for each sample site and then averaged for each fish species to ensure that site‐specific selectivity was maintained.

Finally, we calculated the degree of diet overlap to assess diet similarities among fish species at a site using numerical gut content abundances. Mean proportional abundances were compared pairwise among species using Schoener's similarity index:

$$C=1-\frac{1}{2}\left(\sum \left|P_{x,i}-P_{y,i}\right|\right)$$

where C is Schoener's similarity index metric, and *P*_*x*,*i*_ and *P*_*y,i*_ are the proportions of diet item *i* in the gut of species *x* and *y*, respectively (Schoener, 1970). This index ranges from 0 to 1 with values of 0 indicating no diet overlap and values of 1 indicating a complete overlap of diet items. Schoener's index values higher than 0.6 or lower than 0.4 are generally considered ecologically relevant (Childs et al., 1998; Muth & Snyder, 1995; Wallace Jr, 1981).

### 2.4 Statistical analyses

Statistical analyses were conducted using R version 3.4.3 (R Core Team, 2017). We used non‐metric multidimensional scaling (NMDS) with Bray–Curtis distance to examine relationships among fish diet contents by species. NMDS generates an ordination based on a specified number of dimensions and attempts to meet the conditions of a rank similarity matrix (Clarke, 1993). NMDS also produces stress values to quantify the effectiveness of an ordination for pattern analysis, with values below 0.2 considered to be compliant (Clarke, 1993). This method uses ranked distances and is therefore useful for data that fail to meet the assumptions of normality (Clarke & Warwick, 2001; McCune et al., 2002). Pearson's correlations were conducted using NMDS scores from fish diets and the abundance of invertebrate orders and these coefficients were plotted to show the degree of association between fish species and diet items (West et al., 2003).

We also conducted an analysis of similarity (ANOSIM) to test the null hypothesis that there was no difference among the insect assemblages found in the guts of our sampled fishes. ANOSIM produces a test statistic (*R*) that quantifies the differences that are observed between the variables tested. *R* is expressed as a number between 1 and −1, which is interpreted as maximum similarity between groups and maximum dissimilarity between groups, respectively (Clarke, 1993). An *R*‐value of 1 would indicate complete dissimilarity between two groups, an *R*‐value of 0 is interpreted as complete similarity among groups, and a negative *R*‐value suggests that there is more similarity between groups than within groups.

## 3 RESULTS

We processed a total of 471 guts from nine species across 20 sites in the Wyoming Mountain steppe and 12 sites in the Mongolia Mountain steppe (Table 1). The fish collected in the Wyoming consisted of three nonnative trout species (Brook Trout *Salvelinus fontinalis*, Brown Trout *Salmo trutta*, Rainbow Trout *Oncorhynchus mykiss*) and a single native trout species (Yellowstone Cutthroat Trout *Oncorhynchus clarkii bouvieri*), representing a single family: Salmonidae. The Mongolia fish assemblage was more diverse with five native species (Arctic Grayling *Thymallus arcticus*, Sharp‐snouted Lenok *Brachymystax lenok*, Common Minnow *Phoxinus phoxinus*, Russian Weather Loach *Cobitis melanoleuca*, and Siberian Stone Loach *Barbatula toni*), representing four families: Salmonidae, Cyprinidae, Cobitidae, and Nemacheilidae. Of the 471 guts processed, only two guts were empty (0.7%) in Wyoming, while we observed 19 empty guts (8.7%) in fishes collected from Mongolia. Fishes with empty guts were not used for further analysis. We identified 25 families of invertebrates in Mongolia gut samples and 27 families in Wyoming gut samples (Table 2). The three most abundant families in Mongolian fishes were Chironomidae, Hydropsychidae, and Heptageniidae. In Wyoming fishes, Baetidae, Chironomidae, and Brachycentridae were the three most abundant families. Piscivory was extremely rare in our samples, and we did not find any evidence of mammals or amphibians in our diet samples. In Wyoming samples, only two individuals (<1%) had fish present in the gut while in Mongolia there were six individuals (<3%) with fish in their guts.

**TABLE 1:** Summary biological data for fishes used in diet analysis for Wyoming and Mongolia Forest steppe ecoregions.

Region	Species	*n* (empty)	Standard length (mm ± SD)	Mass (g ± SD)
United States	Brook Trout	93 (1)	167.6 ± 40.8	58.8 ± 36.9
	Brown Trout	84 (1)	192 ± 57.7	86.6 ± 56.0
	Cutthroat Trout*	34 (0)	167.1 ± 67.2	72.5 ± 75.6
	Rainbow Trout	44 (0)	183.4 ± 33.0	69.4 ± 30.2
Mongolia	Arctic Grayling*	25 (0)	151.7 ± 96.5	67.9 ± 89.6
	Sharp‐snouted Lenok*	49 (1)	123.8 ± 99.0	65 ± 184.4
	Common Minnow*	66 (2)	61.7 ± 11.0	2.5 ± 1.1
	Russian Weather Loach*	29 (7)	84 ± 14.4	2.7 ± 1.0
	Siberian Stone Loach*	47 (9)	71.5 ± 18.0	3.6 ± 2.1

*Note*: Asterisks (*) indicate native species.

**TABLE 2:** Dietary proportions by amphipod family (or mollusk phylum) from stomachs of fishes collected Wyoming and Mongolian Forest steppe ecoregions.

Order—family	Dietary proportion	
	Mongolia	Wyoming
Amphipoda—Dogielinotidae	—	<0.01
Coleoptera—Chrysomelidae	—	<0.01
Coleoptera—Coleoptera	<0.01	—
Coleoptera—Dytiscidae	<0.01	<0.01
Coleoptera—Elmidae	—	0.042
Coleoptera—Gyrinidae	—	<0.01
Coleoptera—Haliplidae	<0.01	<0.01
Coleoptera—Hydrophilidae	—	<0.01
Diptera—Blephariceridae	<0.01	—
Diptera—Ceratopogonidae	<0.01	—
Diptera—Chironomidae	0.499	0.193
Diptera—Simuliidae	0.034	0.042
Diptera—Syrphidae	—	<0.01
Diptera—Tipulidae	0.012	<0.01
Ephemeroptera—Ameletidae	<0.01	—
Ephemeroptera—Baetidae	0.066	0.218
Ephemeroptera—Caenidae	<0.01	<0.01
Ephemeroptera—Ephemerellidae	—	0.091
Ephemeroptera—Ephemeridae	0.013	—
Ephemeroptera—Heptageniidae	0.109	0.057
Hemiptera—Corixidae	<0.01	<0.01
Hemiptera—Notonectidae	—	<0.01
Hydracarina—Hydracarina	0.040	—
Megaloptera—Corydalidae	—	<0.01
Megaloptera—Sialidae	<0.01	<0.01
Mollusca—Valvatidae	0.013	—
Odonate—Coenagrionidae	<0.01	—
Odonate—Gomphidae	<0.01	—
Plecoptera—Perlidae	0.036	0.037
Plecoptera—Plecoptera	0.012	—
Trichoptera—Brachycentridae	0.015	0.166
Trichoptera—Glossosomatidae	<0.01	—
Trichoptera—Hydropsychidae	0.109	0.026
Trichoptera—Limnephilidae	<0.01	0.074
Trichoptera—Philopotamidae	—	<0.01
Trichoptera—Phryganeidae	—	<0.01
Trichoptera—Polycentropodidae	—	<0.01
Trichoptera—Rhyacophilidae	—	0.011

### 3.1 Diet contents

**FIGURE 2:**
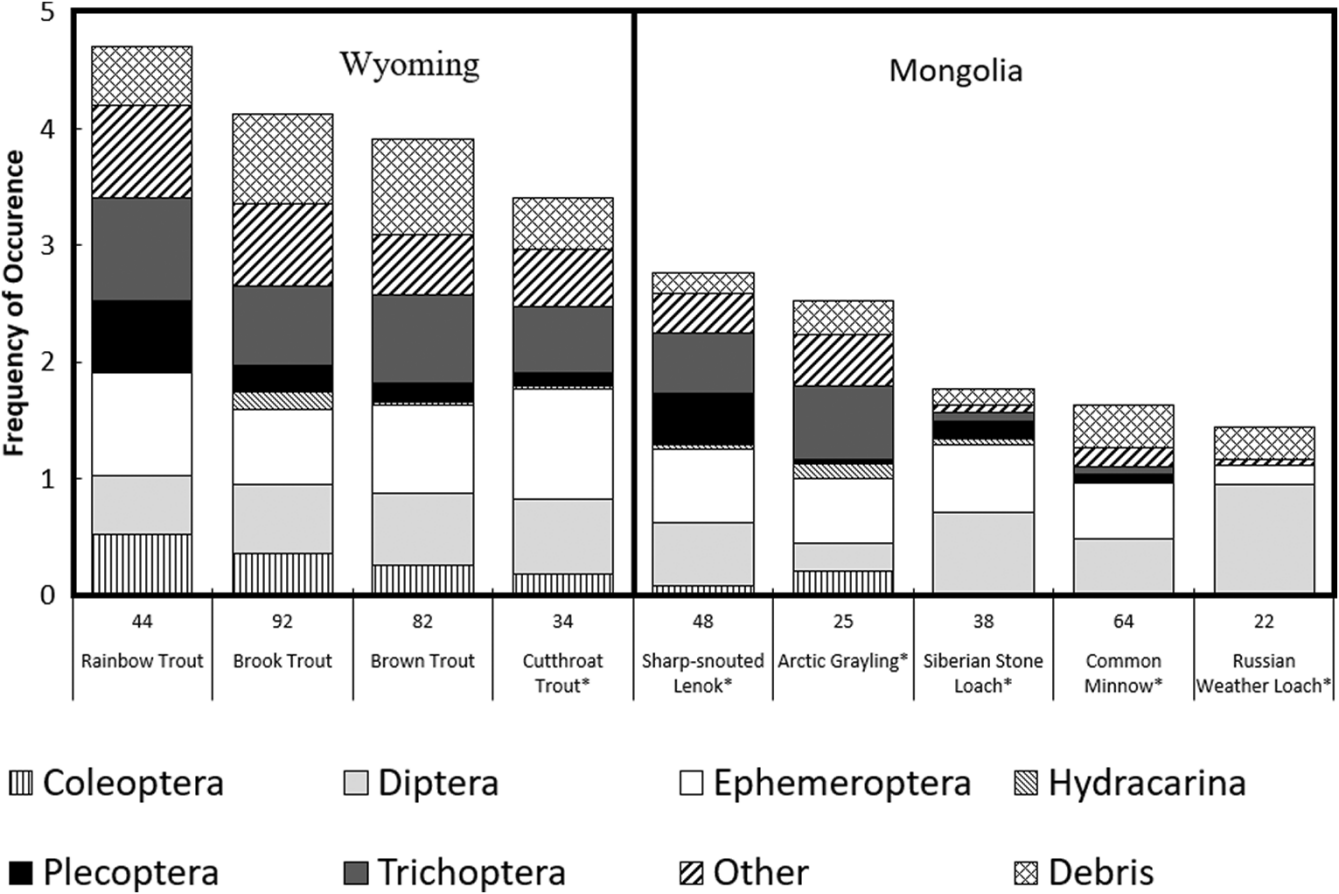
Bar graph displaying the frequency of occurrence for each of the eight diet items that were recorded for all species of fish in Wyoming and Mongolia. The diet items are Coleoptera, Diptera, Ephemeroptera, Hydracarina, Plecoptera, Trichoptera, “other,” and debris.

The frequency of occurrence (FO) of diet items in the guts of collected fishes provides the simplest quantification of diets and is resilient to errors due to diet item condition and digestions (Figure 2). Nonnative salmonids had more diverse diets than native species. The most abundant invertebrate orders observed in diets were Ephemeroptera (56%), Diptera (53%), and Trichoptera (48%); Hydracarina (4%), and Coleoptera (16%) were the least abundant. All Mongolian fishes had lower FO scores than Wyoming fishes. Russian Weather Loach had the highest single FO value of any fish with 94% containing Diptera.

**FIGURE 3:**
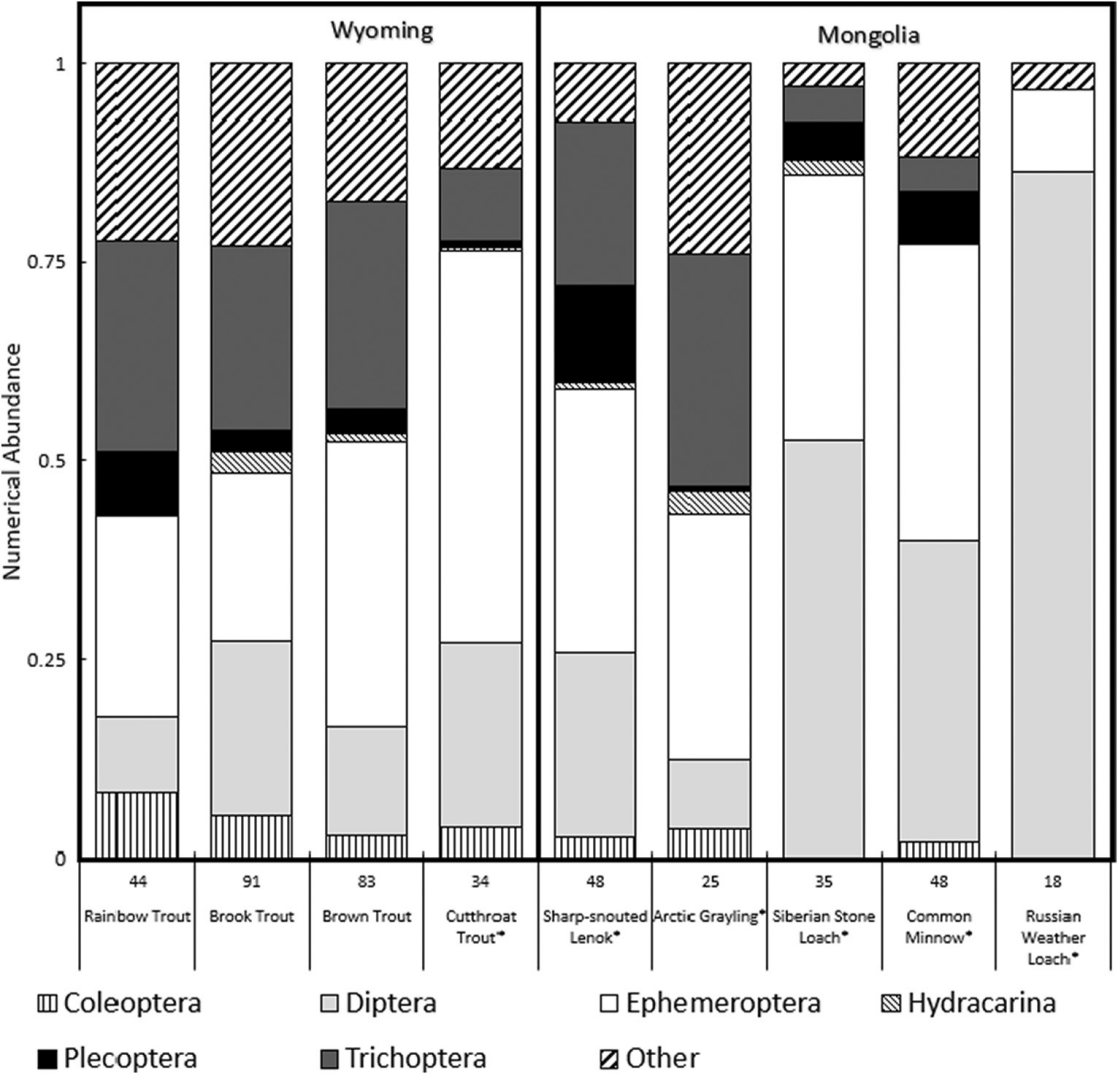
Bar graph displaying the numerical proportion for each of the seven diet items that were recorded for all species of fish in Wyoming and Mongolia. The diet items are Coleoptera, Diptera, Ephemeroptera, Hydracarina, Plecoptera, Trichoptera, and “other.” Debris is removed for this table due to its non‐discrete properties.

When contents were quantified by numerical abundance, we excluded debris from analyses due to its non‐discrete properties (Figure 3). In fishes collected from Mongolia, Diptera (34.8%) and Ephemeroptera (24.2%) were the two most abundant diet item orders. In Wyoming, Ephemeroptera (33.0%) and Trichoptera (21.3%) were most abundant. Gut contents were significantly different among continents, but differences were not strong (ANOSIM; *R* = .23, *p* < .01). The largest difference in assemblages was driven by the proportion of Diptera between the Wyoming (17.2%) and Mongolia (34.9%). Wyoming fishes all had relatively similar diet contents with some variation, but these differences were not significant (ANOSIM; *R* = .031, *p* = .014). Conversely, we detected differences among guts for species collected in Mongolia, these differences were significant but not strong (ANOSIM; *R* = .11, *p* = .001).

### 3.2 Diet selectivity

**FIGURE 4:**
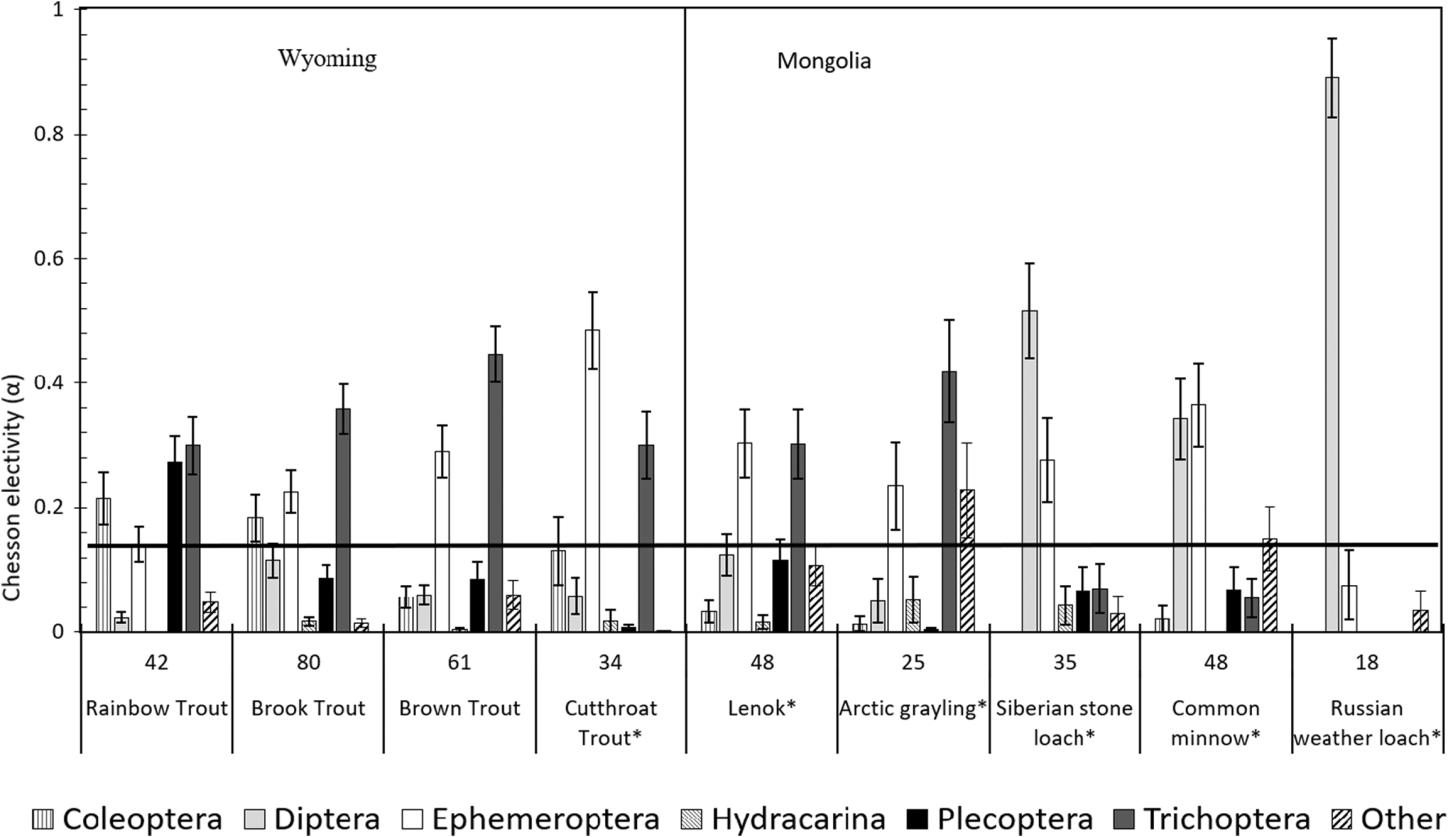
Bar graph displaying the selectivity values for each of the seven diet items that were recorded for all species of fish in Wyoming and Mongolia. The diet items are Coleoptera, Diptera, Ephemeroptera, Hydracarina, Plecoptera, Trichoptera and “other.” There is a line that is placed horizontally across the figure that represents organisms consuming items at amounts equal to what is found in the environment. Values above that line represent positive selection and below that line represents avoidance.

The average environmental abundance proportions for diet items in each ecoregion results in a comparison of the invertebrate assemblages across ecoregions (Table 3). Chesson's α selectivity analysis was calculated using site‐specific abundances for diet items for each individual and displayed several patterns among species (Figure 4). Russian Weather Loach had the highest selectivity of all species and its diet differed greatly from all other fishes. In Wyoming fishes, selection was moderately variable among species, but all fishes selected Trichoptera, and none selected for Diptera. Cutthroat Trout had the highest selectivity of all Wyoming species and selected for Ephemeroptera. In Mongolian fishes, selection varied greatly among species. Sharp‐snouted Lenok and Arctic Grayling both had similar diet preferences, both selecting Ephemeroptera and Trichoptera. Common Minnow and Siberian Stone Loach both selected Diptera and Ephemeroptera and differed greatly in their selection of diet items belonging to the “Other” category as well as Common minnow completely avoiding Hydracarina. Among continents, Brown Trout and arctic graying diets were very similar but we did not see a great deal of difference between any salmonids.

**TABLE 3:** Average numerical environmental abundance proportions for invertebrates in each sample region.

Invertebrates	Region	
	Wyoming	Mongolia
Coleoptera	0.0325	0.0127
Diptera	0.5139	0.4624
Ephemeroptera	0.2011	0.2329
Hydracarina	0.0292	0.0191
Plecoptera	0.0281	0.0510
Trichoptera	0.0688	0.0348
Other	0.1264	0.1871

*Note*: Invertebrates were grouped by order, unless only a single group in that order was collected. Orders that represented <1% of the total number of diet items among all species were grouped into the “Other” category.

### 3.3 Diet overlap

Schoener's similarity index results suggested significant diet overlaps (C > 0.60) among many of the species we sampled (Table 4). All Wyoming fishes displayed significant within and among taxa diet overlap (Table 4). Among Wyoming fishes, Rainbow Trout had the highest overlap scores with both other nonnative trout species (0.84) and the lowest overlap score with the native Cutthroat Trout (0.62). For Mongolian fishes, we found an even mix of positive, negative, and neutral overlaps (ranging for 0.22–0.84). The highest overlap we detected was between Siberian Stone Loach and Common Minnow (0.84), and the lowest score was between Russian Weather Loach and Arctic Grayling (0.22).

**TABLE 4:** Schoener's similarity matrix (C) for all species combinations in Wyoming and Mongolian Forest steppe ecoregions.

	Arctic Grayling*	Sharp‐snouted Lenok*	Common minnow*	Russian weather loach*	Siberian stone loach*	Brook trout	Brown trout	Rainbow trout
Sharp‐snouted Lenok*	**0.71**							
Common Minnow*	0.58	**0.77**						
Russian Weather Loach*	*0.22*	*0.37*	0.52					
Siberian Stone Loach*	0.49	**0.69**	**0.84**	**0.66**				
Brook Trout	**0.83**	**0.77**	**0.64**	*0.36*	0.55			
Brown Trout	**0.88**	**0.82**	**0.71**	*0.27*	0.59	**0.82**		
Rainbow Trout	**0.87**	**0.74**	0.6	*0.23*	0.47	**0.84**	**0.84**	
Cutthroat Trout*	**0.67**	**0.77**	**0.79**	*0.37*	**0.65**	**0.71**	**0.76**	**0.62**

*Note*: Values are the result of comparisons of mean numerical proportions of gut contents for each diet item. Scores >0.6 (bold) and <0.4 (italicized) are considered ecologically important and represent high and low levels of diet overlap. Gray boxes represent pairs that did not co‐occur at sample sites. Asterisks (*) indicate native species.

**FIGURE 5:**
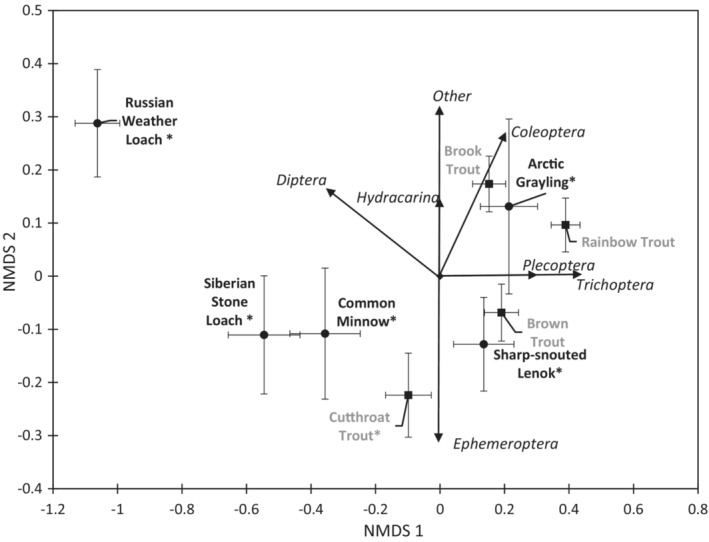
Biplot of an NMDS ordination of fish gut contents by fish species, containing correlation vectors with diet items from samples collected in rivers of Wyoming and Mongolia mountain steppe. Points and bars are the mean and standard error for each species collected. Vectors represent significant correlation vectors for diet items represented with italics. The analysis converged in two dimensions with a stress of 0.17. Gray fonts and black squares represent Wyoming samples, black names and black circles represent Mongolian samples. Fishes that overlap have high dietary overlap. Asterisks (*) indicate native species.

Non‐metric multidimensional scaling used the numerical abundance of invertebrates from the guts of sampled fishes to generate ordinations. The NMDS analysis converged in two dimensions with a stress of 0.17, meeting the threshold for usable pattern analysis. Low standard error values in NMDS (Figure 5) represent low variability in diets (specialists) and large standard errors represent high variability (generalists). Fish species separated on the first NMDS axis by variation in gut contents of Trichoptera, Plecoptera, and Diptera. Fish species separated on the second NMDS axis based on variation in gut contents of Ephemeroptera, Diptera, Coleoptera, and “Other” taxa. Russian Weather Loach gut contents were distinct from all other fishes in the NMDS ordination, driven by its preference for Diptera, with low diet variation. The diet of Wyoming fishes varied only in the relative abundances of several orders of invertebrates and these differences were not significant (ANOSIM: *R* = .023, *p* = .064). Mongolian fishes tended to have higher dietary variation and minor but significant separation in ordination space by taxa (ANOSIM; *R* = .13, *p* = .001). Salmonids in Wyoming and Mongolia appeared to consume similar diet items, but we found significant differences by region (ANOSIM; *R* = .25, *p* = .001). We detected significant differences in diets when comparing Wyoming salmonids to Mongolia non‐salmonid fishes (ANOSIM; *R* = .45, *p* = .001).

Examination of Pearson correlations for invertebrate order abundances in guts with NMDS axis scores uncovered several significant associations (Figure 5). Cutthroat Trout were distinctive from other trout species in the ordination and correlated with Ephemeroptera gut abundance. Brook Trout correlated with Coleoptera gut abundance while Brown Trout and Rainbow Trout did not have strong correlations with gut abundances of any taxa. Mongolian fish species were not as strongly correlated with specific diet abundances as fishes in Wyoming. Arctic Grayling diets were correlated with Plecoptera, Trichoptera, and Coleoptera gut abundances. Sharp‐snouted Lenok diets were not strongly correlated with gut abundances of any taxa and occurred in the same ordination position as Brown Trout. Siberian Stone Loach and Common Minnow diets correlated with Coleoptera gut abundance and Russian Weather Loach correlated with Diptera gut abundance.

## 4 DISCUSSION

Our comparison of the fish assemblages and diets of fishes in heavily stocked rivers of the Wyoming mountain steppe to rivers in northern Mongolia where stocking is absent provides baseline information in Mongolia which can be used to compare the impacts of possible invasions or stocking events in the region. We found that in Wyoming, nonnative species had diets that fit a generalist category with lower levels of selectivity in comparisons with the native cutthroat trout. Cutthroat trout tended to have higher levels of dietary specificity and selectivity, supporting our first hypothesis. Mongolia mountain steppe collections consisted of all native fishes, had higher dietary variability, higher degrees of selectivity, greater variability in Schoener's dietary overlap scores (0.22–0.84), and significant differences in diets, supporting our second and third hypotheses. While salmonids collected in Mongolia and Wyoming had statistically significant differences in their diets, they shared many similarities and displayed high degrees of dietary overlap, as well as having similar selectivity values. The environmental abundance of diet items differed across sites; thus, selectivity provided a useful tool for comparing diets across continents that accounts for site‐specific diet item abundances.

Fish assemblages in the Wyoming mountain steppe were characterized by low species richness, with only four species from a single family (Salmonidae) being collected. The only native species collected was Cutthroat Trout and the additional taxa were nonnative Brook Trout, Brown Trout, and Rainbow Trout. In contrast, fish collections from Mongolia had higher species richness with five native species from four families (Salmonidae, Cyprinidae, Nemacheilidae, and Cobitidae). We found that native species tended to have lower levels of dietary overlap than nonnative species. Nonnative species (Wyoming only) had lower levels of selectivity and diets were more generalist compared to native Cutthroat trout.

Although we found statistically significant differences in numerical salmonid diets among regions, these differences were minor. Piscivory was extremely rare, selectivity preferences were similar, and dietary overlap values among all salmonids were significant. Our collections resulted in many smaller‐bodied fishes, but we did also collect fishes that would be expected to display piscivory. The lack of piscivory may be due to seasonal diet trends that we missed with a single collection event. Diets for all salmonids were dominated by Ephemeroptera, Diptera, and Trichoptera, but Diptera was not positively selected by any salmonid and only neutrally selected by Lenok and Brook Trout. Nonnative salmonids were associated more closely with the Mongolian salmonids than with native Cutthroat Trout in the NMDS ordination. Mongolian salmonids had moderate levels of dietary overlap, small differences in their selection of diet items and distinct separation at sites where they co‐occur, possibly due to resource partitioning—similar to trends found by Olson et al. (2016). Higher FO values and moderate selectivity values in Wyoming salmonids suggest these fishes are dietary generalists compared to Mongolian salmonids (Baker et al., 2014).

Diets in non‐salmonid fishes (Mongolia only) were dominated by Diptera (μ = 59%) and showed high levels of selection for Diptera. It is unlikely that the non‐salmonid fishes in Mongolia would compete with the salmonids due to body size differences. Siberian Stone Loach and Common Minnow had large dietary overlaps, similar selection for diet items, and high overlap in the NMDS ordination. Russian Weather Loach and Siberian Stone Loach occupied distinct microhabitats and were spatially segregated. Russian Weather Loach were restricted to lower elevation sites in our collections, and preferred smaller food items (Copp & Vilizzi, 2004; ROBOTMAM, 1977). These microhabitat preferences likely caused the observed dietary differences and NMDS ordination positions we found for the two species.

Together, low species richness and a high proportion of nonnative species in Wyoming mountain steppe fish assemblages is indicative of invaded ecosystems (Dudgeon et al., 2006; Hermoso et al., 2011; Ross, 1991). In contrast, Mongolian mountain steppe fish assemblages had higher species richness with lower dietary overlap among species and higher levels of selectivity for diet items. Although species richness was low in our Wyoming collections, juveniles may be acting functionally separate based on diets as in other systems (Moyle & Vondracek, 1985). However, the low sample size of juvenile fishes in our study prevented further investigation.

Our comparison of fish diets for Mongolian and the Wyoming mountain steppe regions provided information that native species in Wyoming rivers have conservation concerns from stocked nonnative species, that Mongolian rivers do not currently experience. High abundances of nonnative species and high levels of dietary overlaps in our Wyoming sites is a cause of concern for native Cutthroat Trout and overall system stability (Griffith, 1988; McHugh & Budy, 2006; Peterson et al., 2004). In contrast, fish assemblages characterizing Mongolia mountain steppe rivers were composed of only native species with distinct diets and higher selectivity values, suggesting low probability for intraspecific competition within the two groups of fish we collected (salmonids and non‐salmonids). We recommend a reevaluation of any proposed plans to introduce nonnative salmonids to the area, or if introduction cannot be avoided, to require pre‐ and post‐introduction monitoring of fish assemblages and diets to assess any impacts on native fishes (Jensen et al., 2009; Mercado‐Silva et al., 2008). We also recommend examination of fish diets in both regions during multiple seasons for greater understanding of temporal trends among fishes and to determine how piscivory varies seasonally in these areas.

## AUTHOR CONTRIBUTIONS

**Mario Minder:** Conceptualization (lead); data curation (equal); formal analysis (lead); investigation (equal); software (equal); writing – original draft (lead). **Emily R. Arsenault:** Conceptualization (supporting); investigation (equal); writing – review and editing (equal). **Amarbat Otgonganbat:** Investigation (equal); writing – review and editing (supporting). **Bud Mendsaikhan:** Funding acquisition (supporting); investigation (equal); project administration (supporting); resources (equal); writing – review and editing (equal). **Mark Pyron:** Conceptualization (supporting); funding acquisition (lead); investigation (equal); supervision (lead); writing – original draft (supporting); writing – review and editing (equal).

## FUNDING INFORMATION

This project was funded by an NSF Macrosystem Biology Grant (1442595) to M. Pyron and 10 Co‐PIs.

## CONFLICT OF INTEREST STATEMENT

The authors of this study have no competing interests to disclose.

## Data Availability

The datasets generated during and/or analyzed during the current study are available from Dryad (https://doi.org/10.5061/dryad.rjdfn2zhk).

## APPENDIX A

### Abiotic stream metrics for our sample sites in Wyoming and Mongolian field sites sampled in 2017.

**Table:**

Site	Date	Latitude	Longitude	SSO	AWW (m)	Dis (cm)	MAP (cm)	Elev (m)	Spp
Mongolia forest steppe									
Eg river									
Site ER1	16‐Sep	50.52210	101.43799	4	12	7.9	31	1130	4
Site ER2	17‐Sep	50.56906	101.52899	5	16	7.2	32	1121	5
Site ER3	16‐Sep	50.50471	101.75117	4	10	12.8	32	1102	4
Site ER4	21‐Sep	50.09561	101.59262	4	2	0.4	28	1168	2
Site ER5	22‐Sep	50.31176	101.94098	5	2	0.3	30	1102	4
Delgermörön river									
Site DR1	7‐Sep	50.17475	98.48839	2	1	<0.1	33	1721	3
Site DR2	7‐Sep	50.17598	98.48741	3	4	0.9	33	1719	4
Site DR3	9‐Sep	50.10378	98.60467	4	8	1.8	25	1665	4
Site DR4	10‐Sep	50.12805	98.63965	4	6	1.7	25	1649	4
Site DR5	4‐Sep	49.62554	99.57769	5	30	38.6	19	1326	2
Site DR6	1‐Sep	49.62298	99.68978	5	31	47.3	19	1316	5
Site DR7	2‐Sep	49.63669	99.93284	5	15	7.7	19	1284	7
Wyoming forest steppe									
Bighorn river									
Site BR1	11‐Jul	44.24383	−107.22243	1	3	1.6	27	2709	4
Site BR2	22‐Jul	44.23128	−107.23232	2	4	ND	25	2608	0
Site BR3	12‐Jul	44.20452	−107.23695	2	5	1.1	25	2601	3
Site BR4	21‐Jul	44.20365	−107.24035	2	5	ND	25	2608	2
Site BR5	22‐Jul	44.08428	−107.30853	3	6	ND	25	2341	2
Site BR6	17‐Jul	44.19248	−107.21010	1	1	0.3	25	2643	2
Powder river									
Site PR1	18‐Jul	44.32106	−106.95025	2	2	1.0	46	2243	2
Site PR2	18‐Jul	44.30295	−106.95336	2	2	1.0	46	2281	3
Site PR3	20‐Jul	44.27635	−106.95258	3	3	1.4	49	2364	1
Site PR4	17‐Jul	44.25207	−106.95328	1	1	0.4	51	2406	2
Site PR5	18‐Jul	44.16916	−106.91528	2	2	0.4	44	2353	2
Site PR6	18‐Jul	44.19484	−106.92791	1	1	0.4	44	2473	2
Tongue river									
Site TR1	26‐Jul	44.79442	−107.68860	2	1	0.2	72	2637	1
Site TR2	26‐Jul	44.80872	−107.72080	1	1	0.1	72	2704	1
Site TR3	27‐Jul	44.79861	−107.76511	1	1	0.1	68	2692	2
Site TR4	25‐Jul	44.72168	−107.44931	3	4	1.7	63	2573	2
Site TR5	25‐Jul	44.68661	−107.44595	3	3	1.0	66	2584	2
Site TR6	27‐Jul	44.77088	−107.47100	3	4	1.6	64	2341	2

Abbreviations: AWW, average wetted width (m); Dis., discharge (cm); Elev., elevation; MAP, mean annual precipitation (mm); ND, no data; Spp., number of fish species collected; SSO, Strahler stream order.
